# An analysis of clinical characteristics of rare bilateral medial medullary infarction: An observational study

**DOI:** 10.1097/MD.0000000000038336

**Published:** 2024-06-21

**Authors:** Zhenzhu Hu, Jin Ban, Zhaoying Li, Dongdong Yang, Ke Li, Xuanchao Zhang, Fangfang Hu, Qingqing Li, Wenqi Mao, Yanjing Liang, Dehua Luo, Zhenwei Chen, Hao Chen, Yu Shi

**Affiliations:** a Department of Neurology, XuZhou New Healthy Geriatric Hospital, Xuzhou, Jiangsu, China; b Department of Neurology, The Affiliated Hospital of Xuzhou Medical University, Xuzhou, Jiangsu, China; c Department of Neurology, Hospital of Chengdu University of Traditional Chinese Medicine, Chengdu, China; d Department of Neurology, Xuzhou Cancer Hospital, Xuzhou, Jiangsu, China.

**Keywords:** bilateral medial medullary infarction (BMMI), clinical features, magnetic resonance imaging (MRI), vertebral artery

## Abstract

This study aimed to characterize the risk factors, etiology, clinical manifestations, anatomical characteristics, stroke mechanisms, imaging features, and prognosis of bilateral medial medullary infarction (BMMI). A retrospective analysis was conducted on 11 patients with BMMI who met the inclusion criteria at the Affiliated Hospital of Xuzhou Medical University from January 2013 to January 2023. The patients’ imaging and clinical features were analyzed and summarized. Eleven patients (7 male, 4 female), aged 46 to 62 years, met the inclusion criteria. Common clinical presentations included dysarthria (90.9%), dysphagia (90.9%), quadriplegia (81.8%), and so on. Within 72 hours of onset, 8 cases presented with quadriplegia, 2 cases with hemiplegia, and 1 case without limb paralysis. The main risk factor for BMMI was hypertension, followed by diabetes. “Heart appearance” infarcts occurred in 4 cases (36.4%), while “Y appearance” infarcts occurred in 7 cases (63.6%). Among the patients, 3 had unilateral vertebral artery stenosis or occlusion, 5 had bilateral vertebral artery stenosis or occlusion, 2 had normal vertebral basilar artery, and 1 did not undergo cerebrovascular examination. All patients received standardized treatment for cerebral infarction. The prognosis was poor, with 81.8% of patients having an unfavorable outcome, including 1 death, 9 cases of disability, and only 1 patient achieving self-care ability after recovery. BMMI is more prevalent in males aged 45 to 60 years. The main risk factors are hypertension and diabetes. Atherosclerosis is the primary etiological subtype. The main clinical manifestations are dyskinesia, dizziness, quadriplegia, and dysarthria. The prognosis of BMMI is poor. The specific imaging features of “heart appearance” or “Y appearance” infarcts aid in the diagnosis of BMMI.

## 1. Introduction

Bilateral medial medullary infarction (BMMI) is a unique and uncommon type of brainstem infarction. It is estimated that only approximately 0.5% to 1.5% of all strokes result from medial medullary infarctions (MMIs),^[1]^ making bilateral MMIs even rarer. Magnetic resonance imaging (MRI) serves as the most valuable diagnostic tool and holds significant diagnostic importance in identifying BMMI.

BMMI is characterized by a sudden onset of symptoms and is associated with high rates of disability and mortality. Due to its rarity, there is limited available data regarding the clinical features and prognosis of BMMI. In this study, we reported the clinical data of 11 patients with BMMI and conducted a review of relevant literature to enhance clinicians’ understanding of this disease.

## 2. Patients and methods

### 2.1. Patients

**Figure 1.**
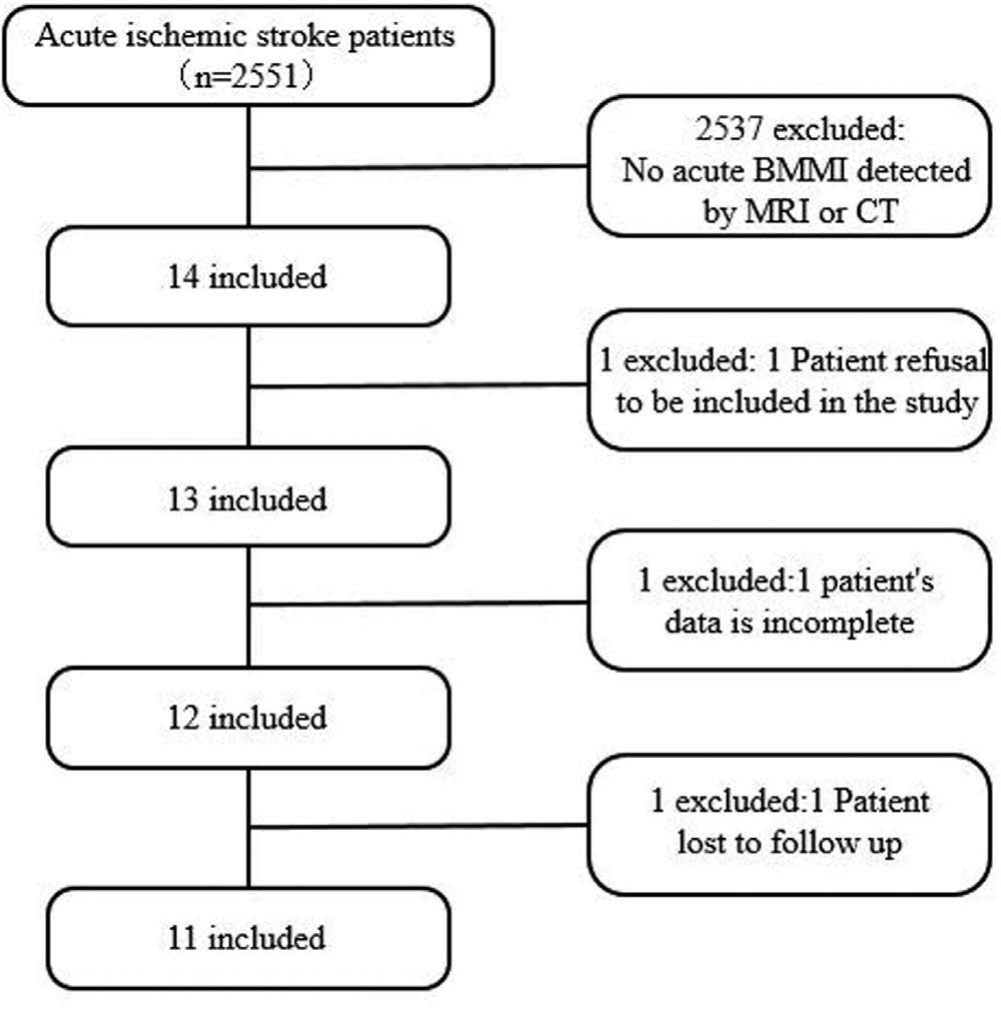
Flow chart of included patients

The study collected data from the past 10 years and included 11 patients with BMMI (Fig. 1). The study was performed with approved protocols and informed consent by the institutional review board of the Affiliated Hospital of Xuzhou Medical University. The authors have also obtained informed consent from the subjects, who all signed an informed consent form for clinical research.

### 2.2. Methods

The detailed demographic and clinical characteristics of the 11 patients are provided in Table 1. In this retrospective study, we analyzed the clinical data of 11 patients with BMMI who were obtained through in-person diagnosis over the past 10 years. The evaluation included an assessment of the patients’ characteristics, clinical features, and prognosis. We also summarized the auxiliary results of the patients, which encompassed brain computed tomography (CT) scans, brain MRI, brain and neck computed tomography angiographies (CTA), and electrocardiogram (ECG).

**Table 1:** The clinical and neuroimaging features of the 11 patients with BMMI.

Case	Age (yrs)/sex	Risk factors	Clinical symptoms	NIHSS/GCS	Lesion location (horizontal)	Vascular status of the VBA	Stroke mechanism	Outcome
1	61/M	Diabetes, smoking, hyperlipidemia	Dysarthria, quadriplegia, deep sensory disturbance	NIHSS = 16, GCS = E3V4M6	Bilateral V + M + D	Severe stenosis in bilateral VA and the BA	LAA	mRS score = 4 GCS = E4V4M6
2	48/M	Diabetes, alcoholism, smoking	Dizziness, dysarthria, quadriplegia, deep sensory disturbance, dysphagia	NIHSS = 18, GCS = E3V4M6	Bilateral V + M + D	Occlusion in the V4 segment of the left VA	LAA	mRS score = 5 GCS = E4V4M6
3	52/M	Hypertension, diabetes, hyperlipidemia, smoking	Dizziness, dysarthria, quadriplegia	NIHSS = 10, GCS = E4V4M6	Bilateral V + M + D	Severe stenosis in bilateral VA and the BA	LAA	mRS score = 4 GCS = E4V4M6
4	46/F	Hypertension, diabetes	Vertigo, nystagmus, dysarthria, quadriplegia	NIHSS = 11, GCS = E4V4M6	Bilateral V + M	vertebrobasilar dolichoectasia with Occlusion in the left VA	VBA	mRS score = 3 GCS = E4V4M6
5	55/M	Hypertension, diabetes, alcoholism, smoking	Dizziness, dysarthria, quadriplegia, dysphagia	NIHSS = 18,GCS = E3V4M6	Bilateral V + M + D	Occlusion in the V4 segment of the left VA	LAA	mRS score = 4 GCS = E4V4M6
6	56/F	Hypertension, diabetes	Dizziness, dysarthria, quadriplegia, nystagmus, dysphagia	NHISS = 19, GCS = E2V1M2	Bilateral V + M + D	Severe stenosis in bilateral VA and the BA	LAA	mRS score = 5, GCS = E2V1M2
7	53/M	Hypertension, diabetes, smoking	Dysarthria, deep sensory disturbance	NHISS = 3, GCS = E4V4M6	Bilateral V + M	Normal findings	SVD	mRS score = 1 GCS = E4V4M6
8	52/F	Myelitis	Dizziness, dysphagia, hemiplegia	NIHSS = 6 GCS = E4V4M6	Bilateral V + M	Normal findings	Other determined etiology	mRS score = 2 GCS = E4V4M6
9	62/M	Hypertension, smoking	Dizziness, quadriplegia, dysarthria, blurred vision, dysphagia	NIHSS = 35 GCS = E1V1M1	Bilateral V + M + D	Severe stenosis in bilateral VA	LAA	mRS score = 5 GCS = E4VTM1
10	52/M	Hypertension, diabetes	Quadriplegia, dysarthria, dyspnea, dysphagia	NIHSS = 29 GCS = E2V1M1	Bilateral V + M + D	/	LAA	mRS score = 5 GCS = E4VTM1
11	55/F	Hypertension	Dizziness, blurred vision, quadriplegia, dysarthria, dysphagia	NIHSS = 7 GCS = E4V4M6	Bilateral V + M	Severe stenosis in bilateral VA	LAA	mRS score = 5 GCS = E1VTM1

D = dorsal part of the medial medulla, F = female, GLC = Glasgow Coma Scale, LAA = large-artery atherosclerosis, M = male, M = middle part of the medial medulla, mRS = modified Rankin scale, NIHSS = National Institute of Health Stroke Scale, SVD = small vessel disease, V = ventral part of the medial medulla, VBA = vertebrobasilar artery, yrs = years.

## 3. Results

### 3.1. Clinical manifestations

The average age of onset for the 11 patients with BMMI was 53.8 ± 4.6 years, ranging from 46 to 62 years. Among the patients, 7 were males (63.6%) and 4 were females (36.4%). Significant medical histories included hypertension in 8 cases (72.2%), diabetes mellitus in 8 cases (72.2%), smoking in 6 cases (54.5%), hyperlipidemia in 2 cases (18.2%), and alcoholism in 2 cases (18.2%). The clinical features observed in the patients included consciousness disturbance in 6 cases (54.5%), dysphagia in 10 cases (90.9%), quadriplegia in 9 cases (81.8%), vertigo/dizziness in 8 cases (72.7%), deep sensory disturbance in 3 cases (27.3%), blurred vision in 2 cases (18.2%), and dyspnea in 1 case (9.1%). Babinski sign was positive on both sides in all patients. Upon admission, the severity of cerebral infarction was assessed using the National Institutes of Health Stroke Scale (NIHSS) and Glasgow Coma Scale. The patients were categorized into 3 grades based on their NIHSS scores: normal or mild stroke (0–4 points) in 1 case (9.1%), moderate stroke (5–15 points) in 4 cases (36.4%), and severe stroke (>15 points) in 6 cases (54.5%). None of the patients received intravenous thrombolysis or intravascular therapy.

### 3.2. Imaging examination

**Figure 2.**
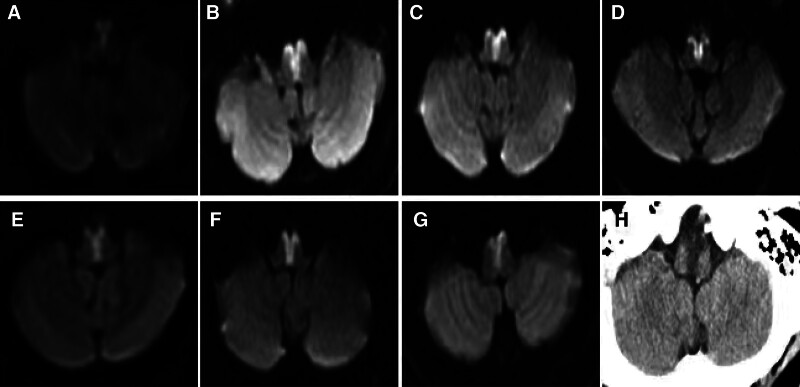
(A–G) Imaging characteristics of the bilateral medial medullary infarction. MRI of the brain using DWI revealed a characteristic high-intensity signal in the bilateral medial medulla oblongata, which often appears as a “heart appearance,” “V” appearance, or “Y” appearance. (H) CT scan revealed “Y” appearance low density image of the medial medulla oblongata. DWI = diffusion-weighted imaging, MRI = magnetic resonance imaging.

Ten patients underwent brain magnetic resonance (MR) scans within 1 week after onset, revealing the presence of lesions in the rostral medulla (Fig. 2). These lesions exhibited hypointensity signals on T1-weighted imaging (T1WI) and apparent diffusion coefficient (ADC), as well as hyperintensity signals on T2-weighted imaging (T2WI), fluid-attenuated inversion recovery (FLAIR), and diffusion-weighted imaging (DWI). However, 1 patient was unable to undergo the MR scan due to his critical condition. In his initial head CT scan, no obvious abnormal signs were detected. However, a follow-up CT scan was performed after 24 hours to monitor the development of the infarct lesion (Fig. 2H).

Among the 11 patients, 10 underwent head and neck CTA, while 1 patient could not complete the vascular examination due to the severity of his condition. The CTA results revealed severe stenosis in bilateral vertebral arteries (VA) in 5 cases, unilateral vertebral artery stenosis or occlusion in 3 cases, normal vertebral basilar artery in 2 cases (Table 1).

### 3.3. Treatment and outcome

The patients included in the study received bedside rehabilitation. All the patients were given dual antiplatelet agents, lipid-lowering agents, etc. Patients with onset < 48 h were given argatroban anticoagulation. The risk factors of cerebrovascular diseases, such as blood pressure, blood glucose levels, and lipid levels, were controlled within the time period of the acute window phase, and the patients with a large cerebral infarction area were given mannitol to alleviate brain edema and strengthen the nursing care. Upon discharge, modified Rankin scale (mRS) score was used to evaluate their prognosis (Table 1) and the patients were divided into 2 grades: good prognosis (0–2 points) and poor prognosis (3–6 points). All the patients had a poor prognosis except for 2.

## 4. Discussion

**Figure 3.**
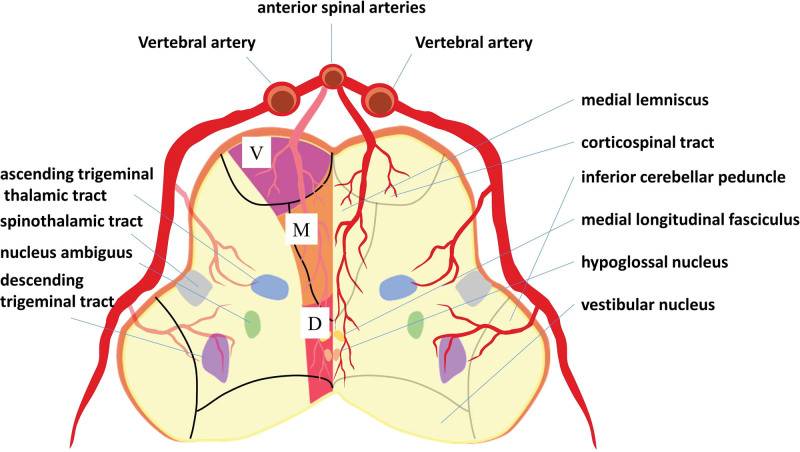
Diagrams of the blood supply of the medulla and its important structures. Schematic diagram showing 4 vascular territories of the rostral medulla and its important structures including 3 portions (V, M, and D).

The medulla oblongata, located at the lowest part of the brainstem, contains various important structures such as the pyramidal tracts, medial colliculus (or colliculus interlacing), and the ipsilateral hypoglossal nerve. In terms of anatomical arrangement, these structures can be observed from the ventral (front) to the dorsal (back) side, as depicted in Figure 3. BMMI predominantly affects the dorsolateral medulla. While less prevalent than dorsolateral medulla syndrome, medial medulla infarction can also occur. However, BMMI is a rare condition characterized by severe clinical manifestations, symptoms, and a notably high mortality rate.

BMMI tends to be more prevalent among individuals aged 45 to 60 years, with a higher incidence in males compared to females, as supported by previous studies.^[2,3]^ In our study, patients under the age of 60 accounted for 81% of the cases. Fan Hu et al^[3]^ reported 15 cases of BMMI, of which 60% were under 60 years old, and 80% were male patients, which significantly outnumbered female patients, aligning with our findings. This gender difference may be attributed to factors such as smoking, drinking, obesity, and high work pressure in men, which can accelerate the occurrence and progression of cerebrovascular diseases.

Hypertension is the most common cause of BMMI in this study consistent with previous reports. The prevalence of hypertension is typically higher in males than females, which may contribute to the disparity in incidence rates of hypertension and diabetes between genders. Additionally, males under 60 years of age with hypertension and diabetes often have a family history and experience early onset of these conditions. Furthermore, due to busy work and other factors, the daily monitoring and management of blood pressure and blood sugar are often neglected.

Diabetes mellitus was another primary risk factor associated with BMMI in our study. Among the patients, there were 8 individuals with type 2 diabetes. This suggests a close relationship between BMMI and type 2 diabetes, where small vessel lesions and vertebral artery lesions resulting from diabetes may contribute to the pathogenesis of BMMI. Our study revealed that BMMI is more commonly observed in middle-aged male patients with untreated hypertension and/or hypertension diabetes. For young and middle-aged patients with hypertension and diabetes, it is crucial to prioritize regular cerebrovascular examinations and evaluations. This approach allows for the early detection of atherosclerosis and enables the timely initiation of anti-atherosclerosis therapy. By implementing such measures, efforts can be made to delay the onset and progression of cerebral atherosclerosis.

**Figure 4.**
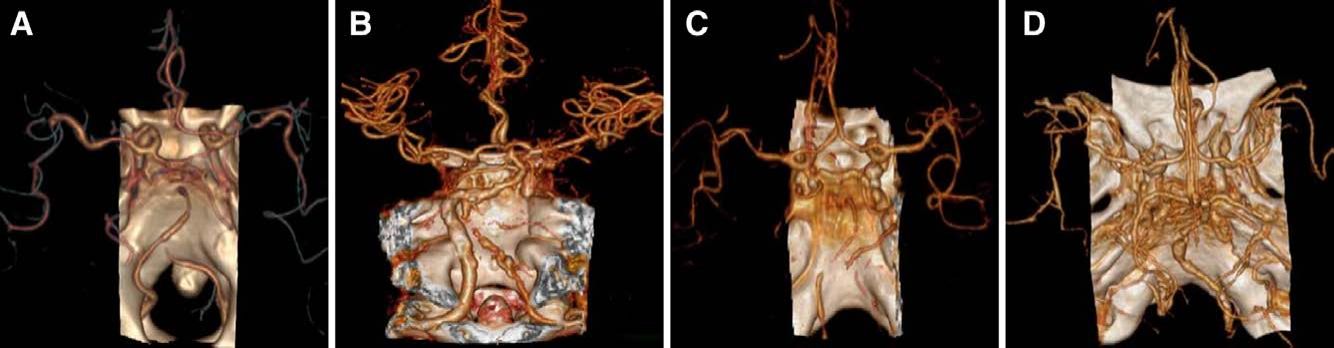
Computed tomography angiography (CTA) scans of the head and neck CTA scans of the head and neck showed severe stenosis or occlusion of the vertebral artery, basilar artery, or posterior cerebral artery.

CTA scans of the head and neck showed severe stenosis or occlusion of the vertebral artery, basilar artery, or posterior cerebral artery (Fig. 4). As shown in Figure 4A and C, the main cause of stroke related to BMMI is large-artery atherosclerosis (LAA). The second f cause of stroke related to BMMI is small vessel disease (SVD). We also observed 1 patient with vertebrobasilar dolichoectasia (Fig. 4D), which is a condition characterized by elongation and dilation of the vertebrobasilar arteries. However, it is important to note that other studies have reported additional risk factors for BMMI. These include Fabry disease,^[4]^ cerebral embolism,^[3]^ fusiform vertebral artery aneurysms,^[5]^ vertebral artery dissection,^[6]^ and Takayasu arteritis.^[7]^

The clinical manifestations, severity, and prognosis of patients with BMMI can vary significantly. In all patients, the onset of symptoms was acute, and treatment was initiated within 72 hours. Prior to the onset of the disease, some patients experienced precursor symptoms such as dizziness, gait unsteadiness, and nausea/vomiting, which worsened over time. One patient initially developed hemiplegia, which progressed to tetraplegia after 2 days. This progression may be attributed to uneven damage to the bilateral pyramidal tracts during the onset of the disease.

In our study, the majority of BMMI cases presented with dysarthria, dysphagia, and limb paralysis during acute onset, with facial function typically unaffected. We observed a significant correlation between the vascular territories, ischemic features observed on MRI, and neurological examination findings associated with medullary infarctions, which is consistent with previous research results.^[8]^ Motor weakness was the most common manifestation of BMMI, consistent with previous reports.^[2,3]^ Quadriplegia or sensory dullness in all 4 limbs may be the initial symptom of BMMI.^[9]^ Two patients in our study experienced respiratory failure and required tracheostomy placement during their initial hospitalization. Pseudobulbar palsy was present in both patients. Respiratory disturbances appeared to result from impairment of the medial medullary reticular formation, as well as the nuclei of ambiguous, retroambigualis, and tractus solitaries.^[10]^ Additionally, limb weakness contributed to increased bed-rest time for the patients. In patients with pseudobulbar palsy and weakened sputum clearance, there is a higher likelihood of sputum accumulation in the throat. Failure to remove the sputum promptly can lead to hypoventilation, carbon dioxide retention, and even pulmonary infections. During hospitalization, 6 out of the 11 patients developed complications of pulmonary infection. It is recommended to elevate the head of the bed by at least 30 inches to prevent aspiration pneumonia and worsen the condition. Caution should be exercised when administering nasal feeding fluids to avoid such complications.

The incidence of progressive stroke in patients with BMMI was found to be high. In our study group, 5 cases (45.4%) experienced a progressive stroke, with the peak time of symptom progression occurring between the 2nd and 6th day after onset. It can be challenging to diagnose bilateral bulbar infarction based solely on clinical manifestations, as it needs to be differentiated from conditions such as Guillain-Barre syndrome, brainstem encephalitis, myasthenia gravis, hypokalemic periodic paralysis, and other diseases.

Brain MRI plays a valuable role in facilitating early diagnosis. In our study, we observed a higher incidence of heart type medullary infarction than previously reported. This finding prompts an important discussion about diagnostic techniques. Simple CT scans, while useful in many settings, are prone to misdiagnosis and missed diagnoses in cases of medullary infarction. The limitations of CT in detecting early or small infarctions are well-documented, largely due to its lower sensitivity in identifying acute ischemic changes in the brainstem and medulla. Given these limitations, our study underscores the necessity of employing MRI, and specifically DWI sequences, in the diagnostic protocol. DWI has proven to be exceptionally sensitive in detecting acute ischemic strokes, including heart type medullary infarctions. The high signal intensity in the lesion area observed in DWI sequences during the super early stages of infarction provides a critical advantage. This capability allows for more accurate and timely diagnosis, which is paramount in guiding appropriate clinical interventions and improving patient outcomes. The increased incidence rate observed in our study could, therefore, be attributed to the enhanced diagnostic capabilities of MRI compared to CT. This technological advancement allows for the detection of cases that might have previously been overlooked, especially when only CT scanning was employed. MRI of the brain using DWI revealed a characteristic high-intensity signal in the bilateral medial medulla oblongata, which often appears as a “heart appearance,”^[11]^ “V” appearance, or “Y” appearance. The “Y” appearance indicates complete involvement of the anteromedial territory, while the “heart appearance” suggests partial involvement of the anterolateral territory. The specific shape of the lesion, such as “V” or “heart,” may be related to the location and severity of the infarction as well as the affected vascular territory.

In our study, we observed that the “heart” shaped infarcts were predominantly located in the upper part of the medulla oblongata, while the “Y” shaped infarcts were mainly located in the middle part of the medulla oblongata. Additionally, 2 patients had involvement of both the upper and middle parts of the medulla oblongata, and they presented with respiratory failure requiring tracheostomy. This suggests that when the lesions in the medulla oblongata involve a wider range, respiratory failure is more likely to occur.

The medulla oblongata receives its blood supply from the vertebrobasilar system. The vascular territories within the medulla oblongata are divided into anteromedial, anterolateral, lateral, and posterior regions based on their blood supply. The characteristic “heart appearance” seen on imaging results from infarctions involving the bilateral anteromedial and anterolateral territories. The primary blood supply to this region is derived from the vertebral and anterior spinal arteries.

The origin and pattern of the anterior spinal artery can vary significantly. The distance between the origins of the 2 branches (left and right) forming the anterior spinal artery, as well as their distance from the vertebrobasilar junction and the origin of the posterior inferior cerebellar artery, exhibit high variability.^[12]^ For example, when the anterior spinal artery emanates from the left or right unilateral vertebral artery, a blockage at the site of origin of this single anterior spinal artery trunk can lead to the development of BMMI.^[12,13]^ This condition has been described as “The Airpod Sign.”^[14]^ Variations in the anatomy of the vertebral artery are not uncommon, and understanding these anatomical variations is crucial for both anatomy studies and clinical practice. Occlusion of the anterior spinal artery, when the medial medulla oblongata is supplied unilaterally, can result in BMMI.^[15]^ It is important to note that severe stenosis or loss of a unilateral vertebral artery can also occur in the general population. High-resolution MRI can aid in distinguishing between normal anatomical variations and the responsible vessel in such cases.

After admission, all 11 patients received standardized treatment for cerebral infarction. Some patients with severe symptoms were transferred to the intensive care unit (ICU), while others, despite their severity, chose to discontinue treatment and were discharged. None of the patients received thrombolysis upon admission. A total of 81.8% of the patients were discharged with a MRS score of 3 or higher, indicating a poor overall prognosis for BMMI despite the small size of the infarcts. In this study, 2 patients died in the acute stage. One of them developed severe pneumonia and respiratory failure. After being hospitalized in the Neurological Intensive Care Unit for 5 days, the family opted to discontinue treatment, and the patient was discharged. Unfortunately, the patient later passed away at home. The other patient also experienced severe pneumonia and eventually died due to secondary multiple organ dysfunction syndrome (MODS). Our findings indicate that patients with a “heart appearance” infarction can develop bulbar paralysis and respiratory failure. Most patients remain in a long-term coma.

The patients in this group had prolonged hospital stays, except for those who chose to discontinue treatment. The longest hospitalization duration was 63 days, but the prognosis did not improve significantly. Progressive stroke was observed in 5 patients, all of whom had poor outcomes. The characteristic pattern of disease progression within 2 to 6 days after onset suggests that the underlying pathological mechanism of BMMI is closely related to in situ thrombosis caused by atherosclerosis, which may be suitable for thrombolytic therapy and interventional thrombolysis. However, none of the patients in this group received such treatments, which can be analyzed based on 2 main reasons. First, it may be attributed to a lack of awareness and understanding of the disease among clinicians, resulting in delayed diagnosis. Second, due to the critical nature of the disease, patients were often admitted to the ICU without timely improvement of brain MRI and digital subtraction angiography (DSA) examinations. It is important to note that the administration of recombinant tissue plasminogen activator (rt-PA) is approved within 45 minutes after the onset of stroke, and its benefits have been observed up to 4.5 hours poststroke in select patients. Real-world clinical data has shown a trend toward fewer hemorrhagic complications with the use of rt-PA in the extended or unknown time window for ischemic stroke. These findings support the use of rt-PA beyond the initial 4.5-hour window, even in selected patients with posterior circulation stroke. For BMMI patients, who have a poor prognosis with conservative treatment, more aggressive approaches such as thrombolysis or interventional thrombolysis are necessary. In patients with delayed presentation (>4.5 hours) and those with stroke upon awakening, the use of rt-PA in a “super-indication” manner has shown to be safe and effective, reducing mortality and disability rates. However, it is crucial to provide comprehensive explanations and obtain informed consent from patients before applying these interventions.

### 4.1. Limitation

This study has several limitations that should be considered. First, it was a single retrospective observational study conducted at a single institution, which limits the generalizability of the findings to a broader population. The results may be influenced by institutional practices and patient characteristics specific to that setting. Second, the small number of participants in this study may limit the statistical power and precision of the results. A larger sample size would provide more robust findings and enhance the reliability of the study. Third, the cerebrovascular examination performed on patients in this group primarily relied on CTA, which has limitations in detecting arterial wall abnormalities and branch artery diseases. The use of techniques such as DSA and high-resolution MRI could provide more detailed information on these structures. Future research should aim to incorporate these advanced imaging modalities to gain further insights into the pathophysiology of BMMI. With the ongoing advancements in imaging technology, we anticipate that future studies will uncover additional findings that can contribute to a deeper understanding of this condition.

## 5. Conclusion

MRI scans of BMMI brain lesions show “heart appearance,” or “Y appearance,” due to their particular anatomical location. This feature is helpful for early clinical diagnosis and treatment of such cases. The most common symptom in patients is motor disorder. Two main stroke etiologies of BMMI are LAA and SVD. In cerebrovascular examination, unilateral or bilateral vertebral artery V4 segment lesions. BMMI has a higher disability and mortality rate than general stroke, which requires early identification and more aggressive thrombolysis or thrombolysis.

## Author contributions

**Formal analysis:** Zhenzhu Hu, Dongdong Yang, Qingqing Li, Wenqi Mao.

**Investigation:** Zhenzhu Hu, Jin Ban.

**Writing – original draft:** Zhenzhu Hu, Jin Ban, Zhaoying Li, Ke Li, Xuanchao Zhang, Zhenwei Chen.

**Writing – review & editing:** Zhenzhu Hu, Jin Ban, Zhaoying Li, Dongdong Yang, Fangfang Hu, Yanjing Liang, Dehua Luo, Hao Chen, Yu Shi.

**Resources:** Jin Ban, Xuanchao Zhang, Fangfang Hu.

**Data curation:** Ke Li.

**Supervision:** Yu Shi.

**Visualization:** Yu Shi.
